# Pressure Pain Hyperalgesia Expressed by Topographical Pressure Pain Sensitivity after Cardiac Surgery

**DOI:** 10.3390/life14101233

**Published:** 2024-09-26

**Authors:** Bárbara Segura-Méndez, Álvaro Planchuelo-Gómez, Álvaro Fuentes-Martín, Pascal Madeleine, Ángel L. Guerrero, Yolanda Carrascal, César Fernández-de-las-Peñas

**Affiliations:** 1 Cardiac Surgery Department, University Clinical Hospital, 47005 Valladolid, Spain; barbaraseg@hotmail.com; 2 Imaging Processing Laboratory, Universidad de Valladolid, 47005 Valladolid, Spain; a.planchuelo.gomez@gmail.com; 3 Thoracic Surgery Department, University Clinical Hospital, 47005 Valladolid, Spain; a.fuentesma@saludcastillayleon.es; 4 Department of Health Science and Technology, Aalborg University, 9260 Aalborg, Denmark; pm@hst.aau.dk; 5 Headache Unit, Neurology Department, University Clinical Hospital, 47005 Valladolid, Spain; gueneurol@gmail.com; 6 Department of Medicine, Dermatology and Toxicology, University of Valladolid, 47005 Valladolid, Spain; 7 Department of Surgery, Ophthalmology, Otorhinolaryngology and Physiotherapy, University of Valladolid, 47005 Valladolid, Spain; 8 Department of Physical Therapy, Occupational Therapy, Physical Medicine and Rehabilitation, Universidad Rey Juan Carlos (URJC), 28922 Alcorcón, Spain; cesar.fernandez@urjc.es

**Keywords:** cardiac surgery, postoperative pain, pressure pain maps

## Abstract

Backgrounds: We aim to evaluate changes in pressure pain sensitivity before and after cardiac surgery using topographical sensitivity maps utilizing a pressure algometer. Methods: Pressure pain thresholds over 17 thoracic points and 4 distant pain-free points were assessed in 70 patients (women: 29, age: 67.5 years), before and at 1, 3, and 7 postoperative days. Thoracic topographical pressure pain sensitivity maps were calculated at all follow-ups. Postoperative pain was recorded at each follow-up on a numerical pain rate scale. Results: Postoperative pain intensity decreased from 6.4 (SD 1.0) on the first postoperative day to 5.5 (SD 1.9) on the third and to 4.5 (SD 1.7) on the seventh day (*p* < 0.001). The mixed-model ANOVA revealed that the lowest pressure pain thresholds were observed one day after surgery, increased slightly during follow-up, and were lower at the xiphoid process. Significant negative correlations between postoperative pain intensity and pressure pain thresholds were observed at each time point in thoracic measures (all, *p* < 0.01), but not with pressure pain thresholds from distant pain-free areas. Conclusions: Postoperative pain after cardiac surgery can be objectively quantified using algometry. Pressure pain hyperalgesia was associated with the intensity of postoperative pain.

## 1. Introduction

Multiple studies have tried to identify potential risk factors associated with postoperative pain after cardiac surgery, but the subjectivity of most pain measurement scales acts as a limiting factor in this setting. Pressure algometry stands as a straightforward, non-intrusive, and objective modality that yields a biophysical metric for gauging pressure pain sensitivity. Indeed, the evaluation of mechanical hyperalgesia through the computation of pressure pain thresholds (PPTs) ranks among the most prevalent quantitative sensory examinations for appraising modified nociceptive pain transduction in individuals with chronic pain [1]. The presence of pressure pain hyperalgesia after minor or major surgery has been previously documented [2,3]. These studies have discerned the emergence of extensive pressure pain hyperalgesia as an indicator of nociceptive processing alteration post-surgery.

It is possible that the occurrence of pressure pain hyperalgesia will be implicated in post-surgical pain following cardiac procedures. Nevertheless, a narrative review showed that pressure pain sensitivity is heterogeneously distributed across the body’s regions of interest [4] and depends on the topographical area under evaluation. To the best of our knowledge, no previous study has investigated topographical pressure pain sensitivity maps after cardiac surgery.

### Hypothesis and Aim of the Study

The primary aims of the current study were to investigate changes in pressure pain sensitivity before and after cardiac surgery and to determine if such alterations are heterogeneous by constructing topographical pain maps. A secondary aim of the study was to analyze if sensitivity to pressure pain after cardiac surgery was associated with postoperative pain intensity.

## 2. Materials and Methods

### 2.1. Participants

A prospective, observational study of a single-center cohort was executed. Patients older than 18 years undergoing cardiac surgery, with or without extracorporeal circulation, from January to June 2021, were eligible to be included after written informed consent. The investigation adhered to the STROBE (Strengthening the Reporting of Observational Studies in Epidemiology) directives [5]. The study protocol (identification number PI19-1493) received full approval from both the local Institutional Research Review Committee and the Clinical Research Ethics Committee.

Preoperatively, we excluded patients under 18 years of age, those who underwent urgent or emergency surgery, and those presenting with cognitive impairments or linguistic barriers precluding assessment feasibility. After a preoperative assessment, patients who were unable to participate in postoperative measurement acquisition due to intubation or sedation were also excluded from the study.

### 2.2. Pain Assessment

An 11-point numerical pain rate scale (NPRS, 0–10) was used to assess the intensity of postoperative pain at one, three, and seven days post-surgery. Pressure pain thresholds (PPTs) were calculated by using a pressure algometer (Pain Diagnosis and Treatment Inc., Great Neck, NY, USA) with a 1 cm^2^ rubber tip. The algometer was calibrated prior to each data record. Pressure was applied perpendicularly to each point at a speed of 1 kg/cm^2^/s. To mitigate intra- and inter-observer variability, recruiters (BS and AF) underwent adequate training before the onset of the study. Three consecutive measurements at each point were collected at every site, separated by at least 30 s, to avoid temporal summation. The mean of the three trials was calculated and expressed in kilopascals (kPa).

**Figure 1:**
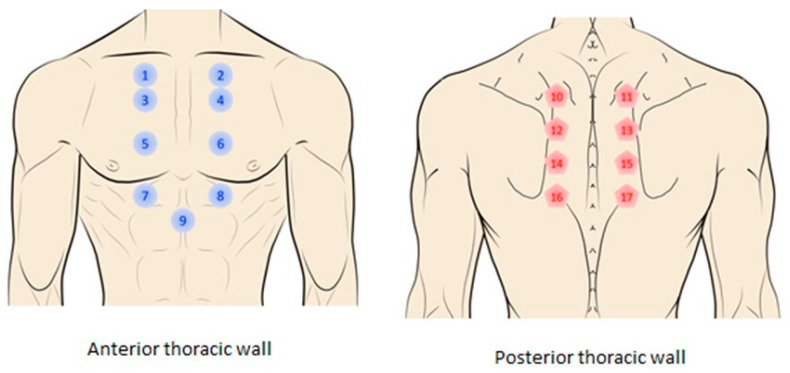
Thoracic points where pressure pain thresholds (PPTs) were assessed for creating topographical pressure pain sensitivity maps: (1) manubrium right side; (2) manubrium left side; (3) 2nd right intercostal space; (4) 2nd left intercostal space; (5) 4th right intercostal space; (6) 4th left intercostal space; (7) 6th right intercostal space; (8) 6th left intercostal space; (9) xiphoid appendix; (10) spinous process T4 left side; (11) spinous process T4 right side; (12) spinous process T6 left side; (13) spinous process T6 right side; (14) spinous process T8 left side; (15) spinous process T8 right side; (16) spinous process T10 left side; (17) spinous process T10 right side.

PPTs were measured preoperative and on one, three, and seven postoperative days at sites shown in Figure 1: (a) 2 cm lateral to both sides of the manubrium (points 1–2); (b) 2 cm lateral to both sides of the sternum at the level of the 2nd (points 3–4), 4th (points 5–6), and 6th intercostal spaces (points 7–8); (c) at the xiphoid process (point 9); (d) 2 cm to both sides of spinous processes of thoracic vertebrae at the level of T6 (points 10–11), T8 (points 12–13), T10 (points 14–15), and T12 (points 16–17). Additionally, PPTs were also assessed bilaterally over the wrist and internal malleolus as distant pain-free areas for evaluating widespread pressure pain sensitivity.

### 2.3. Sample Size 

Sample size was calculated based on the primary aim, with 80% power and a significance level of α equal to 0.05. Preliminary data from 6 individuals showed a pre- to postoperative effect size of 0.46 for PPTs. Therefore, a sample size of 42 subjects was required for the study. Finally, we included 70 patients.

### 2.4. Postoperative Pain Treatment

In all patients, information on analgesic treatment (dose and route of administration) was collected on postoperative days 1, 3, and 7.

### 2.5. Statistical Analysis

IBM SPSS Statistics for Windows, version 25.0 (IBM Corp, Armonk, NY, USA) was utilized for the statistical analysis. The normality of each quantitative variable was assessed by using the Kolmogorov–Smirnov test. Quantitative data are expressed as means (standard deviations). Absolute values and percentages were used to express categorical variables.

For the primary aim of this study, a repeated-measures multivariate analysis of covariance (MANCOVA) using mixed-effect models with time (baseline, one, three, and seven days after) and points (from 1 to 17) as within-subjects variables and with sex and age as covariates, was performed to compare changes of PPT in the thorax post-surgery. A separate MANCOVA using mixed-effect models with time (baseline, one, three, and seven days after) and side (right or left) as within-subjects variables, and with sex and age as covariates, was conducted to compare changes in PPT on distant pain-free areas. Thus, preoperative variables were included as covariates in the main analyses. Corrections for multiple comparisons were performed following a False Discovery Rate (FDR) strategy. Post hoc analyses were conducted with the Bonferroni test.

For the secondary aim, a separate MANCOVA was conducted to evaluate changes in postoperative pain intensity. Additionally, Pearson’s correlation tests were conducted to identify the association between the intensity of postoperative pain and PPTs at each follow-up after cardiac surgery (one, three, and seven days post-surgery).

### 2.6. Topographical Pressure Pain Sensitivity Maps

Mean PPTs over each thoracic location were interpolated using an inverse distance weighted interpolation for graphical purposes and to develop topographical pressure pain sensitivity maps of the thorax at each follow-up period. The inverse distance weighted interpolation involves computing PPT scores to unknown locations by using means from the set of known PPT values and locations [4].

## 3. Results

**Figure 2:**
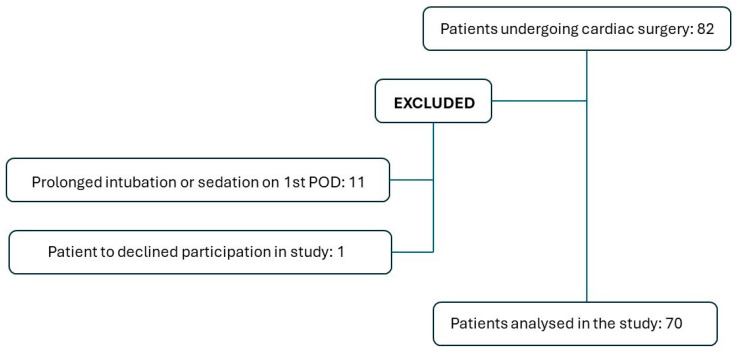
Patient selection process for inclusion/exclusion in the study.

**Figure 3:**
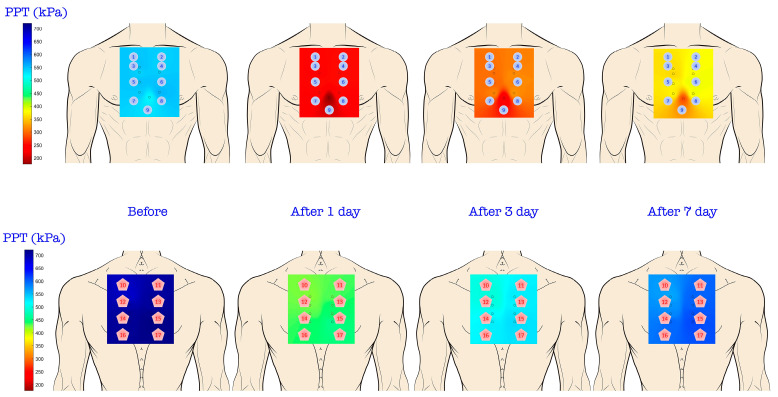
Topographic pressure pain sensitivity maps of the thorax before, and one-, three-, and seven-days after cardiac surgery.

Eighty-two patients who underwent cardiac surgery were eligible for inclusion in this study. Eleven patients were excluded due to prolonged intubation or sedation on the first postoperative day, and one patient declined to participate. Ultimately, data from 70 patients (41.4% women; mean age: 67.5 years) were analyzed (Figure 2). Sternotomy was complete in 90% and a right anterior mini-sternotomy was performed in the remainder. The mini-sternotomy approach was exclusively applied in patients submitted to isolated aortic valve replacement (7 patients). The surgical procedures and preoperative variables are detailed in Table 1 and Table 2, respectively. Figure 3 visualizes topographical pressure pain sensitivity maps before and after cardiac surgery. 

The mixed-model MANCOVA revealed significant time (F = 81.104, *p* < 0.001), sex (F = 30.102, *p* < 0.001), and age (F = 2.386, *p* = 0.015) effects. No significant interaction was observed. Post hoc analyses revealed the following: (1) postoperative pain intensity decreased from 6.4 (SD 1.0) at the first POD to 5.5 (SD 1.9) at the third POD and to 4.5 (SD 1.7) at the seventh POD (*p* < 0.001). The lowest PPTs were observed one day after surgery (*p* < 0.001) and increased slightly on the third (*p* < 0.001) and seventh (*p* = 0.01) postoperative days (Table 3 and the Supplementary Data); (2) the xiphoid process exhibited the lowest PPT at all follow-ups (Table 3) without significant differences between complete sternotomy or mini-sternotomy access; (3) PPTs were higher in posterior than in anterior thoracic locations; (4) females exhibited overall lower PPTs than males in all points at all follow-up periods; and (5) older patients had lower PPTs as compared to younger patients after cardiac surgery. No significant differences were observed between complete sternotomy and mini-sternotomy access in all analyzed PPTs over time. Furthermore, we have analyzed the possible influence of cardiopulmonary bypass (CPB) time in PPTs among the different surgical procedures, excluding significant differences in patients in objective postoperative pain perception attributable to the inflammatory response associated with CPB. 

The mixed-model MANCOVA identified a significant time effect, but not for the side for both distant pain-free points, at the wrist (time: F = 8.256, *p* < 0.001; side: F = 0.678, I confirmP = 0.411) and the internal malleolus (time: F = 11.313, *p* < 0.001; side: F = 0.094, *p* = 0.759): PPTs at the wrist and internal malleolus significantly decreased one day after surgery (*p* < 0.001) but slightly increased at days three (*p* = 0.001) and seven (*p* = 0.03) after surgery (Table 4). There was a significant effect of sex but not of age for PPTs at the wrist (sex: F = 6.814, *p* = 0.01; age: F = 0.703, *p* = 0.405) and at the internal malleolus (sex: F = 8.814, *p* = 0.004; age: F = 1.400, *p* = 0.241): females exhibited lower PPTs than males at all follow-ups.

The repeated-measures MANCOVA revealed a significant time effect for the intensity of postoperative pain (F = 66.124, *p* < 0.001): postoperative pain decreased from 6.4 (SD 1.0) one day after to 5.5 (SD 1.9) three days after (*p* = 0.01) to 4.5 (SD 1.7) seven days after (*p* < 0.001). No effect of sex was observed (F = 1.739, *p* = 0.193). Finally, significant negative correlations between postoperative pain intensity at each time point with PPTs at the thorax (all, *p* < 0.01), but not with PPTs at distant pain-free areas, were observed: one day (−0.399 < r < −0.235); three days (−0.589 < r < −0.323); and seven days (−0.513 < r < −0.302) after surgery. The higher the intensity of postoperative pain, the lower the PPT in the thorax, i.e., the higher the pressure pain sensitivity.

**Figure 4:**
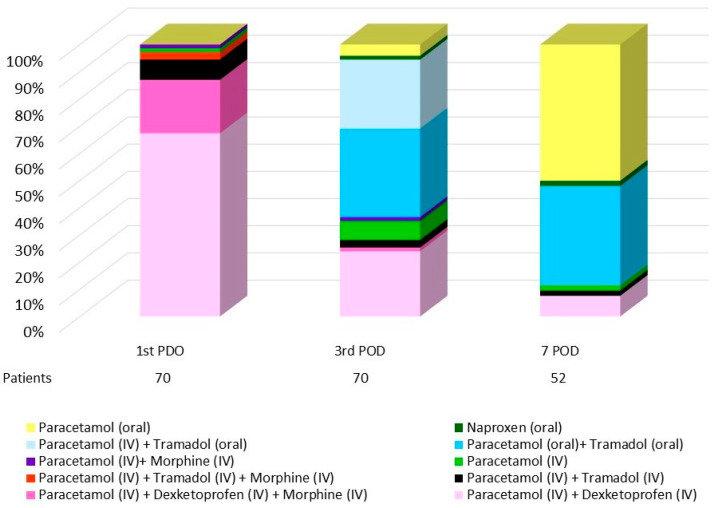
Graphical representation of analgesic treatment and routes of administration throughout the postoperative period.

No preoperative analgesia was used in premedication. Postoperative pain treatment reflects a consistent use of paracetamol and the adaptation of treatment with other analgesics according to patient progress (Table 5). During the first POD, all patients received intravenous analgesia with paracetamol 1 g/8 h, associated with dexketoprofen (50 mg/8 h) (88.6%) or tramadol (50 mg/8 h) (11.4%). An additional 16 patients required treatment with morphine (3 mg/8 h). At the third POD, 98.6% of the patients continued receiving paracetamol (61.4% iv), along with tramadol (43 patients) or dexketoprofen (18 patients). Oral naproxen was administered to one patient. At the seventh POD, paracetamol was administered to 52 patients, along with tramadol or dexketoprofen. Two patients required naproxen treatment (Figure 4).

During postoperative follow-ups, three patients presented with cardiovascular (one with acute myocardial infarction) or respiratory complications (one with pneumonia and one with pneumothorax). We have not observed high levels of postoperative pain or increased analgesic requirements in these patients.

## 4. Discussion

Addressing the complexities of postoperative pain management in cardiac surgery is a complex task due to a confluence of factors, including inadequate pain reporting, patient-specific variability, and the adverse effects of potent analgesics. Scales and questionnaires validated to assess postoperative pain in cardiac surgery are inherently subjective [6,7,8,9,10].

The present study conducted seven days of postoperative monitoring of pain and pressure pain sensitivity following cardiac surgery. This is the first study investigating topographical pain sensitivity maps in subjects after cardiac surgery. The hypothesized outcomes were confirmed, showing the development of generalized pressure pain hyperalgesia in the thorax and widespread pressure pain sensitivity in distant pain-free areas from the first day after cardiac surgery. Further, pressure pain hyperalgesia was positively associated with the intensity of postoperative pain.

The primary aim of this study was to describe changes in sensitivity to pressure pain after cardiac surgery. Therefore, we developed for the first time topographical pain sensitivity maps of the thorax in this population. Topographic pain sensitivity maps disclosed a predominantly homogeneous sensitivity to pressure pain across both the anterior and posterior thoracic regions. Nevertheless, the maps revealed that the xiphoid process showed the lowest PPT and heightened sensitivity to pressure pain, irrespective of the surgical approach employed (complete sternotomy vs. mini-anterior right sternotomy). The xyphoid is cut in half in a median sternotomy, resulting in two mobile, innervated structures. However, xyphoid sensitivity remained higher after the mini-sternotomy approach, potentially attributable to the presence of endothoracic drainage. Thus, the anterior thoracic region demonstrated greater sensitivity, manifesting lower PPTs compared to the posterior region. This finding aligns with expectations, as muscular areas (posterior thorax) typically register higher PPT than bone or joint regions (anterior thorax) [4]. 

Generalized thoracic pressure pain hyperalgesia was observed after cardiac surgery, showing a significant decrease in PPT one day post-procedure, with progressive recovery over the subsequent seven days. In line with our findings, Mazzeffi et al. also reported the lowest pain thresholds at 24h post-surgery [11]. However, it should be noted that PPT did not return to preoperative levels seven days post-surgery. It would be anticipated that any surgical intervention would lead to generalized hyperalgesia in the days following due to the inherent damage associated with the procedure. Cardiac surgery induced immediate generalized hyperalgesia across the entire thorax, largely due to a peripheral mechanism, as the surgery impacts all thoracic segments. We also found widespread hyperalgesia at distant pain-free points, suggesting a role of central sensitization. The sensitivity to pressure pain in distant pain-free areas followed a similar pattern to that in the thorax, i.e., the lowest PPTs were observed one day post-cardiac surgery, with progressive recovery in the days following. These results suggest that the surgical procedure could act as a peripheral driver for the development of acute hyperalgesia to pressure pain in patients undergoing cardiac surgery.

Females exhibited higher pressure pain sensitivity than males at all points and all follow-ups. Evidence supports that females are more susceptible to pain, perceive pain as more intense, and are at a higher risk of developing chronic pain than males [12,13], with biological, cognitive, and social factors accounting for these differences [14]. However, although PPTs were lower in females, the temporal pattern of pressure sensitivity was the same for both sexes; a decrease on the first day post-cardiac surgery with progressive increases in the days following.

Finally, we found a higher susceptibility to pain in older individuals compared to their younger counterparts. The notion that older adults exhibit a higher sensitivity to pressure pain than young adults is corroborated by the existing literature [15]. However, there was considerable methodological heterogeneity in studies examining the effect of age on pain sensitivity. Yet, despite older patients demonstrating lower PPT than younger patients, topographical pain sensitivity maps adhered to the same temporal pattern post-cardiac surgery.

The secondary objective was to ascertain if pressure pain sensitivity is associated with the presence of acute postoperative pain. We observed that a higher sensitivity to thoracic pressure pain correlated with a higher intensity of postoperative pain across all follow-ups. This observation suggests that postoperative pain could potentially drive the hyperalgesia to pressure pain in the thorax via peripheral, but not central, mechanisms. While achieving adequate pain control during the immediate postoperative period following cardiac surgery is complex, our study was unable to determine that patients with lower PPT had worse pain management. Given that postoperative pain can be associated with neuropathic, muscular, and visceral components, it remains unclear whether early management of postoperative pain is crucial for preventing the development of altered nociceptive processing in patients undergoing cardiac surgery. Future studies should, therefore, concentrate on this aspect to enhance the management of postoperative pain.

### Limitations

Although this is the inaugural study investigating topographical pressure pain sensitivity maps in patients undergoing cardiac surgery, several limitations should be acknowledged. Firstly, this study includes patients from a single center and with a relatively small sample size, which may limit the representativeness of the population and the generalizability of the results. Secondly, we did not characterize postoperative pain; thus, we are unaware of the features, e.g., musculoskeletal or neuropathic, of the peripheral drive. Thirdly, the assessment of PPT the day after cardiac surgery could be biased by the surgical procedure itself and the effects of anesthesia. However, the fact that the lowest PPTs were observed immediately post-procedure would exclude a potential anesthetic effect. Fourthly, the patients excluded due to sedation on the first postoperative day may represent a selection bias as they may be more susceptible to postoperative complications and, consequently, to experiencing postoperative pain. Fifthly, although efforts have been made to minimize variability through algometry training techniques, there may be a certain degree of variability among the evaluators. Finally, we did not evaluate psychological aspects or beliefs in relation to surgery, variables that could act as confounding factors in pain sensitivity.

## 5. Conclusions

Postoperative pain in cardiac surgery can be objectively quantified using algometry, based on the demographic and clinical characteristics of the patient. The objective quantification of postoperative pain could allow for the design of appropriate treatment strategies and the evaluation of outcomes based on the objective measurement provided by algometry for each patient group.

## Figures and Tables

**Table 1:** Surgical procedures, cardiopulmonary bypass and aortic clamp times (in minutes).

Surgical Procedure	*n* (%)	CPB Time (min) Mean (SD)	Aortic Clamp Time (min) Mean (SD)
Valve repair or replacement Isolated valve 2 valves 3 valves	36 (51.4)	113.2 (36.5)	86.6 (29.6)
CPBG	20 (28.6)	88.1 (35.5)	58.6 (20.8)
Combined procedures 1 valve replacement + CABG 2 valve replacement + CABG	6 (8.6)	141.8 (51.6)	113 (51.3)
Bentall and Bono procedure	4 (5.7)	124.5 (23.7)	101 (18.9)
Cardiac tumour resection	1 (1.4)	89	53
Aorta replacement	2 (2.9)	125.5 (28.7)	85 (34)
Congenital + 1 valve surgery	1 (1.4)	127	65

CPB: cardiopulmonary bypass; CABG: coronary artery bypass graft.

**Table 2:** Descriptive preoperative variables.

Variable	*n* (%)
Smoking	4 (5.7)
Arterial hypertension	35 (50)
Diabetes Mellitus	16 (22.9)
Dyslipemia	35 (50)
Stroke	3 (4.3)
Renal failure	6 (8.6)

**Table 3:** Mean (standard deviation) of pressure pain thresholds (kPa) measured ad each analyzed point over the time.

Point	Preoperative	1st POD	3rd POD	7 POD
1	549.2 (166.7)	250.05 (58.8)	304.1 (68.6)	362.8 (88.2)
2	549.2 (186.3)	255.0 (49.0)	308.9 (68.6)	382.45 (68.6)
3	539.35 (176.5)	245.15 (49.0)	304.1 (58.8)	357.95 (78.4)
4	544.25 (176.5)	255.0 (53.9)	318.7 (68.6)	382.45 (73.5)
5	549.2 (166.7)	255.0 (78.4)	299.1 (58.8)	372.65 (93.1)
6	549.2 (176.5)	255.0 (49.0)	313.8 (63.7)	387.35 (73.5)
7	544.25 (176.5)	250.05 (49.0)	313.8 (68.6)	372.65 (78.4)
8	554.1 (176.5)	250.05 (53.9)	323.6 (73.5)	387.35 (78.4)
9	519.75 (186.3)	176.5 (39.2)	220.65 (58.8)	284.4 (58.8)
10	706.1 (186.3)	431.5 (98.05)	524.65 (176.5)	598.2 (145.1)
11	681.55 (186.3)	421.7 (107.9)	495.2 (152.0)	563.9 (142.2)
12	706.1 (186.3)	441.3 (107.9)	519.75 (156.9)	598.2 (145.1)
13	701.2 (176.5)	426.6 (98.05)	500.15 (147.1)	573.7 (142.2)
14	715.9 (196.1)	441.3 (98.05)	529.55 (152.0)	608.0 (145.1)
15	711.0 (181.4)	431.5 (98.05)	514.85 (147.1)	578.6 (145.1)
16	720.8 (186.3)	446.2 (107.9)	534.45 (147.1)	612.9 (145.1)
17	711.0 (181.4)	441.3 (98.05)	524.65 (152.0)	598.2 (142.2)

POD: Postoperative day; 1: manubrium right side; 2: manubrium left side; 3: 2nd intercostal right space; 4: 2nd left intercostal space; 5: 4th right intercostal space; 6: 4th left intercostal space; 7: 6th right intercostal space; 8: 6th left intercostal space; 9: xiphoid appendix; 10: spinous process T4 left side; 11: spinous process T4 right side; 12: spinous process T6 left side; 13: spinous process T6 right side; 14: spinous process T8 left side; 15: spinous process T8 right side; 16: spinous process T10 left side; 17: spinous process T10 right side.

**Table 4:** Mean (standard deviation) of pressure pain thresholds (kPa) in distant pain-free areas of the cohort.

Point	Preoperative	1st POD	3rd POD	7 POD
Right wrist	666.8 (186.3)	343.2 (107.9)	431.5 (132.4)	495.2 (122.6)
Left wrist	666.8 (196.1)	363.8 (127.5)	456.1 (137.3)	509.9 (127.5)
Right Leg	696.3 (196.1)	480.5 (166.7)	529.5 (161.8)	549.2 (156.9)
Left Leg	691.4 (98.05)	490.35 (166.7)	534.4 (171.6)	549.2 (166.7)

POD: Postoperative day.

**Table 5:** Postoperative analgesic treatment. Number of patients receiving analgesic drugs over study time.

Drug (Dosage)	1st POD *n* (%)		3rd POD *n* (%)		7 POD *n* (%)	
	IV	Oral	IV	Oral	IV	Oral
Paracetamol (1 g/8 h)	70 (100)	0 (0)	43 (61.4)	26 (37.1)	6 (8.6)	46 (65.7)
Dexketoprofen (50 mg/8 h)	62 (88.6)	0 (0)	18 (25.7)	0 (0)	4 (5.7)	0 (0)
Tramadol (50 mg/8 h)	4 (5.7)	4 (5.7)	2 (2.9)	41 (58.6)	1 (1.4)	19 (27.1)
Morphine (3 mg/8 h)	16 (22.9)	0 (0)	2 (2.9)	0 (0)	0 (0)	0 (0)
Naproxen (400 mg/12 h)	0 (0)	0 (0)	0 (0)	1 (1.4)	0 (0)	2 (2.9)

POD: Postoperative day; IV: intravenous.

## Data Availability

The data are available.

## Supplementary Materials

The following supporting information can be downloaded at: https://www.mdpi.com/article/10.3390/life14101233/s1, Supplementary Data. Mean (standard deviation) of pressure pain thresholds (kPa) measured ad each analyzed point over the time. (POD: postoperative day) according to surgical approach (complete sternotomy (CS) vs. mini anterior right sternotomy (MS)).
