# Supplementary material for: Pressure Pain Hyperalgesia Expressed by Topographical Pressure Pain Sensitivity after Cardiac Surgery
Source: Life (Basel). 2024 Sep 26;14(10):1233. doi: 10.3390/life14101233 (PMC11508688; doi:10.3390/life14101233)
Supplement: Supplementary file 1 [file life-14-01233-s001.zip › life-3213811-supplementary.pdf]

life

MDPI

Supplementary Data. Mean (standard deviation) of pressure pain thresholds (kPa) measured ad each analyzed point over the time. (POD: postoperative day) according to surgical approach (complete sternotomy (CS) vs. mini anterior right sternotomy (MS)).

Point	Preoperative		1st POD		3rd POD		7 POD	
	CS	MS	CS	MS	CS	MS	CS	MS
1	516.2 (144.7)	548.1 (182.4)	251.8 (68.3)	265.6 (94.4)	297.6 (72.6)	295.8 (90.8)	357.8 (90.9)	341.0 (86.8)
2	503.1 (165.1)	577 (207.2)	253.6 (50.03)	258.8 (83.2)	304.9 (64.9)	323.6 (107.1)	381.05 (63.6)	356.6 (80.8)
3	542.9 (171.6)	612.5 (256.4)	249.4 (52.6)	253.5 (101.4)	305.9 (73.9)	303.0 (93.8)	370.0 (80.6)	317.6 (97.7)
4	546.5 (180.9)	640.7 (252.8)	257.2 (52.8)	274.4 (76.2)	322.8 (69.8)	329.4 (90.6)	395.9 (74.9)	356.6 (86.2)
5	525.4 (153.1)	549.5 (199.0)	266.06 (90.2)	255.8 (113.1)	301.5 (74.2)	279.6 (102.6)	374.5 (94.8)	332.0 (111.0)
6	586.6 (158.6)	600.8 (219.0)	258.8 (83.2)	259.6 (53.2)	311.8 (64.5)	315.8 (92.1)	390.2 (67.8)	346.3 (94.5)
7	546.7 (174.1)	632.5 (252.1)	256.3 (51.7)	244.0 (64.6)	318.9 (73.5)	310.2 (92.3)	385.6 (81.9)	339.6 (86.5)
8	553.0 (190.9)	664.0 (236.4)	263.0 (55.3)	283.5 (81.8)	327.1 (62.8)	338.4 (73.8)	399.6 (80.2)	335.3 (96.7)
9	485.1 (165.9)	525.3 (217.4)	173.1 (39.2)	168.5 (26.3)	218.3 (59.5)	221.3 (29.6)	282.6 (65.6)	269.6 (71.9)
10	658.9 (194.4)	815.6 (194.6)	430.6 (109.5)	463.5 (98.7)	594.7 (164.7)	507.8 (118.3)	586.4 (151.09)	520.6 (49.6)
11	649.3 (176.9)	774.1 (201.4)	426.3 (109.2)	478.1 (121.0)	477.5 (139.5)	519.0 (128.2)	565.11 (150.1)	506.6 (75.7)
12	667.1 (197.5)	812.00 (186.9)	440.4 (114.6)	474.1 (99.5)	497.1 (143.4)	516.8 (109.9)	595.1 (151.4)	490.0 (60.82)
13	660.2 (185.09)	787.0 (196.0)	430.5 (104.2)	472.00 (111.2)	479.7 (142.8)	527.5 (103.6)	572.1 (142.9)	513.3 (92.9)
14	676.4 (202.7)	799.1 (193.8)	443.5 (104.3)	485.8 (100.6)	510.6 (141.0)	534.3 (99.8)	610.8 (150.3)	513.0 (75.6)
15	673.04 (188.7)	799.00 (191.9)	438.6 (108.5)	460.8 (105.3)	495.8 (137.3)	537.3 (106.6)	580.3 (145.7)	506.3 (87.3)
16	682.5 (195.6)	825.1 (202.8)	449.8 (114.5)	479.1 (109.6)	517.5 (142.9)	532.5 (90.1)	614.9 (152.7)	524.0 (74.6)
17	675.07 (189.3)	795.6 (200.5)	446.8 (107.0)	479.3 (99.4)	497.7 (142.2)	551.3 (102.9)	592.2 (142.5)	528.6 (69.7)

POD: Postoperative day; 1: manubrium right side; 2: manubrium left side; 3: 2nd intercostal right space; 4: 2nd left intercostal space; 5: 4th right intercostal space; 6: 4th left intercostal space; 7: 6th right intercostal space; 8: 6th left intercostal space; 9: xiphoid appendix; 10: spinous process T4 left side; 11: spinous process T4 right side; 12: spinous process T6 left side; 13: spinous process T6 right side; 14: spinous process T8 left side; 15: spinous process T8 right side; 16: spinous process T10 left side; 17: spinous process T10 right side.
